# Predictive Factors of Capsular Contracture in Prepectoral Direct-to-Implant Breast Reconstruction and its Surgical Approach

**DOI:** 10.1055/a-2505-7591

**Published:** 2025-03-11

**Authors:** Si Youn Kim, Se Won Oh, Eun Jung Yang, Seung Yong Song, Dong Won Lee

**Affiliations:** 1 Department of Plastic and Reconstructive Surgery, Institute for Human Tissue Restoration, Yonsei University College of Medicine, Seoul, Republic of Korea

**Keywords:** breast reconstruction, implant, capsular contracture

## Abstract

**Background** Prepectoral direct-to-implant (DTI) is a common implant-based breast reconstruction method used for patients with breast cancer, although patients often present capsular contracture as a common complication. This study aimed to investigate the causes and surgical outcomes of capsular contractures in patients with breast cancer who underwent prepectoral DTI.

**Methods** The medical records of 392 patients (472 breasts) who underwent prepectoral DTI between August 2019 and July 2022 were retrospectively reviewed. Comparative and multivariate analyses were performed to identify risk factors for capsular contracture. The outcomes of patients who underwent surgical procedures were analyzed.

**Results** Of the 472 breasts enrolled in the study, 47 (9.9%) showed capsular contracture. Multivariate analysis revealed that patient age, seroma, rippling, and postmastectomy radiotherapy were independent correlating factors for capsular contracture in prepectoral DTI. Partial capsulectomy was performed on 18 breasts with capsular contracture, which resolved in 88.9% of cases. The mean follow-up period was 14.4 months.

**Conclusion** Age, seroma, rippling, and radiotherapy were independent correlating factors for capsular contracture in prepectoral DTI. Further, partial capsulectomy is recommended as a treatment option to improve results. A better understanding of the causes and surgical outcomes of capsular contracture on prepectoral DTI will help reduce capsular contracture and eventually lead to better outcomes in breast cancer reconstruction.

## Introduction

Breast cancer is one of the most common malignancies in the female population, 1 and treatment regarding breast cancers involves mastectomy and immediate reconstruction using implants or autologous tissue. Two-stage reconstruction using tissue expander placement followed by implant insertion was once considered the most commonly used method in breast reconstruction using implants. 2 However, with the advancement in skin flap viability during mastectomy and improvement in skin- and nipple-sparing techniques in mastectomy, 3 reconstruction techniques have evolved from two-step reconstruction to the direct-to-implant (DTI) technique, which is a one-step reconstruction, involving immediate insertion of a breast implant after mastectomy. 4

Over the years, breast reconstruction methods have evolved into more efficient and less invasive techniques. The prepectoral technique has become a popular alternative to traditional subpectoral approaches in implant-based breast reconstruction. Key advantages include a reduced risk of breast animation deformities and less discomfort from avoiding pectoralis major muscle elevation. Also, there are no significant differences in postoperative complications, such as infection, skin flap necrosis, or capsular contracture, between prepectoral and subpectoral methods. 4 5 6 7 8 Consequently, the prepectoral DTI technique is gaining traction as a reliable option in breast reconstruction.

Capsular contracture is a common complication in implant-based breast reconstruction. Various studies have investigated potential causes, including implant type, placement plane, use of acellular dermal matrix (ADM), history of chemotherapy and radiotherapy, and the impact of postoperative infections or biofilms. 7 8 9 10 11 12 Despite this, the exact mechanism and contributing factors remain unclear, as findings are often inconsistent due to small sample sizes, differing reconstruction methods, and the lack of matched control groups across studies. Our study aimed to investigate the causes and surgical outcomes of capsular contracture in patients undergoing prepectoral DTI. We focused specifically on prepectoral DTI interventions and the factors influencing capsular contracture. Additionally, we analyzed the surgical outcomes of patients who developed capsular contractures and required further procedures. Through this analysis, we hope to provide guidelines for predicting, treating, and preventing capsular contracture in prepectoral DTI patients, ultimately leading to more personalized and improved reconstructive surgery.

## Methods

### Study Design and Population

**Fig. 1:**
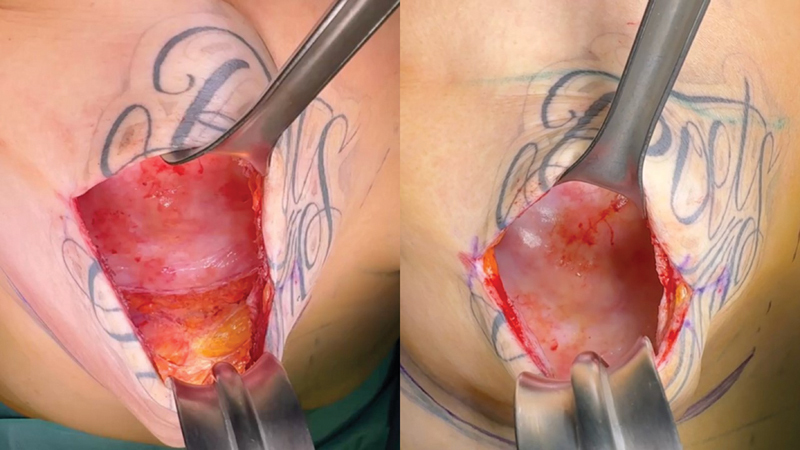
A case of a 51-year-old female, with a history of bilateral breast augmentation, skin-sparing mastectomy, implant change, and radiotherapy. Partial capsulectomy was performed, mainly focusing on the inframammary fold where capsular contracture was noticeable in this patient. Figures during the procedure; before (left) and after partial capsulectomy (right).

Patients who underwent prepectoral DTI (robot-assisted DTI included) between August 2019 and July 2022 at XXX Hospital of the XXX University College of Medicine were enrolled in the study. Our study design was approved by the Hospital Institutional Review Board (IRB approval number 2023-2301-001). To create a homogenous patient population, patients diagnosed with metastatic disease and with less than a 6-month follow-up period were excluded from the study. We analyzed each patient's clinicopathological features, including demographics, TNM (tumor, nodes, and metastases) stage, intraoperative findings (mastectomy type), neoadjuvant and adjuvant therapies, and postoperative complications. Pre- and postoperative clinical photographs were acquired and reviewed. The capsular contracture in this study referred to the degree of grades III (a breast firm to touch that appears distorted) and IV (a breast hard and painful to touch that appears distorted) outcomes according to the Baker–Spear classification system. 13 Surgical infection was defined according to the Centers for Disease Control and Prevention (CDC) surgical site infection definition criteria, which involves infection occurring within 30 or 90 days after the operative procedure involving deep soft tissues of the incision. Hematoma was defined as an incidence of intervention due to its cause, such as negative drain insertion or surgical intervention. Therefore, we examined the association between capsular contracture and these factors. Furthermore, a surgical intervention was performed for patients who experienced discomfort in daily life due to capsular contracture and wished for surgical correction, the decision to proceed with surgery was based on the patient's subjective discomfort rather than the Baker–Spear classification criteria. The surgical results of these groups were analyzed. Among the patients who underwent surgical treatment, informed consents were obtained from participants who agreed to provide pre- and postoperative photographs ( Figs. 1 2 3 ).

**Fig. 2:**
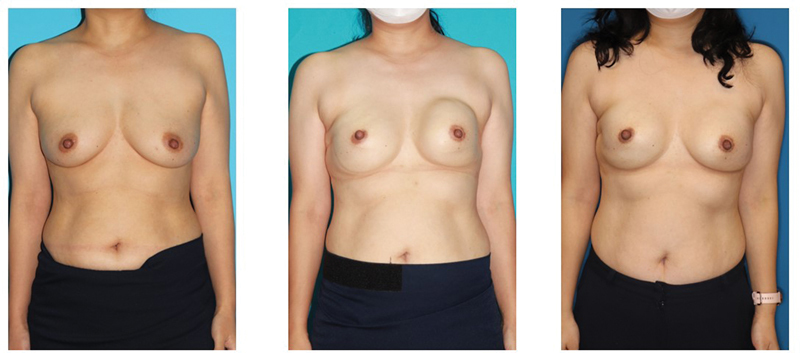
A case of a 40-year-old female, with a history of bilateral prepectoral direct-to-implant (DTI). The figure shows breasts previous to the first implant insertion (left), 31 months after the first implant insertion with noticeable capsular contracture on left side of the breast (middle), and at the 8-month follow-up after capsulectomy was performed (right).

**Fig. 3:**
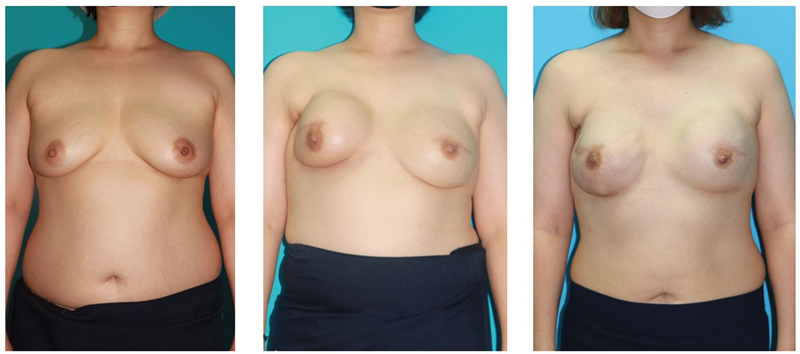
A case of a 41-year-old female, with a history of bilateral prepectoral direct-to-implant (DTI) and radiotherapy. The figure shows breasts previous to the first implant insertion (left), 15 months after the first implant insertion with noticeable capsular contracture on right side of the breast (middle), and the 2-year follow-up after implant change and capsulectomy was performed (right).

### Surgical Techniques

Following mastectomy, a sizer implant was placed to assess the appropriate volume of the breast implant. Indocyanine green was injected to assess the viability of the mastectomy skin flap. After the surgeon determined the volume of the breast implant, it was completely covered with human ADM (MegaDerm graft; L&C Bio Corp., Seoul, Korea) and fixed to the pectoralis major muscle. Complete coverage with ADM was achieved in all patients and a smooth cohesive mammary gel implant was used. The ADM-covered implant was positioned in position by the surgeon, and after placing it in the right position, it was fixed over the pectoralis muscle by fixing the ADM onto the pectoralis muscle with sutures. Sutures were placed at four points on the upper part of the implant to the pectoralis muscle, securing the implant's position. The suture was done from the medial to the lateral side.

Among patients with capsular contracture, during postoperative follow-up, only those patients who were willing to undergo surgery due to its discomfort or appearance underwent surgical correction. An incision was made along the previous incision to remove the implant. The condition of the capsule was examined by the operator and capsulectomy was performed on relatively rigid and pathologically visible capsules as could be seen in Fig. 1 . After partial capsulectomy on relatively visible capsules, resolvement of capsular contracture was noticeable by the operator as the pocket returned to original shape and place during the operation. If the skin flap was too thin, ADM was grafted after capsule removal. Once all pocket operations were completed, a new implant of the appropriate size was reinserted and surgery was completed.

### Statistical Analysis

Continuous variables are expressed as mean ± standard deviation, and nominal variables are expressed as frequency (%). Comparative analyses were performed using the chi-square test and Student's *t* -test. The significance of the differences between groups was assessed using a log-rank test. Multivariate analysis was performed to identify risk factors for capsular contracture using a Cox proportional hazards model. Statistical significance was set at *p* -values <0.05.

## Results

### General Characteristics of Patients

The medical records of 410 patients (a total of 512 breasts) were retrospectively reviewed. To create a homogenous patient population, patients diagnosed with metastatic disease ( *n* = 3) and who were lost during the follow-up period (<6 month follow-up period) were excluded ( *n* = 15) from the study. Therefore, 392 patients and (472 breasts) were retrospectively reviewed. Table 1 shows the demographic and clinicopathological characteristics of the prepectoral group. The mean follow-up period was 20.0 ± 13.9 months, and the patients had an average age of 47.6 ± 9.4 years. The average body mass index (BMI) of the patients was 23.2 ± 3.4 kg/m ^2^ , and the average specimen weight and implant sizes were 396.1 ± 179.0 g and 309.4 ± 100.6 mL, respectively.

**Table 1:** Clinical characteristics, complications and adjuvant therapy concerning cancer

Variable	Mean ± SD
Age, years	47.6 **±** 9.4
BMI, kg/m ^2^	23.2 **±** 3.4
Specimen weight, g	396.1 **±** 179.0
Implant size, mL	309.4 **±** 100.6
Mastectomy type	
Nipple-sparing mastectomy	361 (76.5%)
Skin-sparing mastectomy	82 (17.4%)
Total mastectomy	29 (6.1%)
Stage	
0	111 (23.5%)
IA	152 (32.2%)
IB	2 (0.4%)
IIA	92 (19.5%)
IIB	38 (8.1%)
IIIA	21 (4.4%)
IIIC	4 (0.8%)
Preventive	52 (11.0%)
Complications	
Infection	11 (3.0%)
Capsular contracture	47 (9.9%)
Seroma	24 (5.1%)
Hematoma	28 (5.9%)
Implant rupture	13 (3.6%)
Exposure	12 (2.5%)
Rippling	49 (13.6%)
Malposition	23 (4.8%)
Skin flap necrosis	27 (5.7%)
Adjuvant therapy	
Neoadjuvant CTx	65 (13.7%)
Post CTx	57 (12.1%)
Pre RTx	3 (0.6%)
Post RTx	78 (16.5%)
Anti-EST Tx.	146 (30.9%)
Target Tx.	21 (4.4%)

Abbreviations: anti-EST, anti-estrogen therapy; BMI, body mass index; CTx, chemotherapy; RTx, radiotherapy; SD, standard deviation.

All data are expressed as mean ± sd or *N* (%).

Nipple-sparing mastectomy was performed in 361 breasts (76.5%), skin-sparing mastectomy in 82 (17.4%), and total mastectomy in 29 (6.1%). Regarding postoperative complications, 47 breasts (9.9%) had capsular contractures, 24 (5.1%) showed signs of postoperative seroma, and 11 patients (3.0%) had surgical site infections. Rippling was observed in 54 breasts (11.4%). Approximately 30% of the patients underwent anti-hormonal therapy; 65 breasts (13.7%) underwent neoadjuvant chemotherapy, and 57 (12.1%) received postoperative chemotherapy. Postoperative radiation therapy was administered to 78 breasts (16.5%; Table 1 ).

### Determining Correlating Factors of Capsular Contracture

A multivariate analysis was performed and adjusted for the following confounding factors: age, BMI, tumor stage, mastectomy type, complications, and adjuvant therapies. The analysis showed that age (OR = 0.95, [95% CI: 0.91–0.98], *p* = 0.002), incidence of seroma (OR = 3.57, [95% CI: 1.46–8.62], *p* = 0.004), incidence of rippling (OR = 0.34, [95% CI: 0.08–0.71], *p* = 0.011), and postoperative radiation therapy (OR = 5.29, [95% CI: 1.63–18.28], *p* = 0.007) were independent correlating factors of capsular contracture ( Table 2 ). Higher age and incidence of rippling were independent correlating factors for a lower risk of capsular contracture, whereas the incidence of seroma and postoperative radiotherapy were independent factors leading to capsular contracture. However, due to the association between thicker capsule presence and less pronounced rippling, it is challenging to assert that rippling incidence affects capsular contracture, despite what the analysis indicates.

**Table 2:** Determining risk factors of capsular contracture

Variable	OR	2.50%	97.50%	*p* -Value
Age, years	0.95	0.91	0.98	0.002
BMI, kg/m ^2^	1.11	1.00	1.18	0.132
Stage				
0	(Ref)			
I	1.31	0.44	3.72	0.458
II	10.49	4.40	26.99	0.147
III	31.11	7.63	142.00	0.214
Preventive	3.32	1.24	9.11	0.018
Mastectomy type				
Nipple-sparing mastectomy	(Ref)			
Skin-sparing mastectomy	0.44	0.19	0.98	0.052
Total mastectomy	1.09	0.34	3.24	0.776
Complications				
Infection	2.59	0.71	8.86	0.134
Seroma	3.57	1.46	8.62	0.004
Hematoma	0.10	0.05	0.23	0.141
Rupture	2.16	0.68	6.76	0.185
Rippling	0.34	0.08	0.71	0.011
Malposition	3.62	0.15	46.86	0.330
Necrosis	0.32	0.07	1.21	0.123
Infection	2.59	0.71	8.86	0.144
Adjuvant therapy				
Neoadjuvant CTx	0.19	0.05	0.60	0.137
Post-CTx	0.22	0.07	0.60	0.155
Post-RTx	5.29	1.63	18.28	0.007
Anti-EST	0.77	0.36	1.56	0.444
Target	0.45	0.05	1.70	0.233

Abbreviations: Anti-EST; anti-estrogen therapy; BMI, body mass index; CTx; chemotherapy; RTx, radiotherapy.

### Management of Capsular Contracture

Among the 47 breasts diagnosed with capsular contracture, a partial capsulectomy was performed in 18 breasts, and contractures resolved in 88.9% of the cases during an average follow-up of 14.4 months ( Figs. 2 and 3 ). Relatively visible and rigid capsules on the lateral side of the breast were observed in 16.7% of the cases, whereas 77.8% of cases showed rigid, visible capsules mainly on the lower side. One case (5.6%) showed rigid, visible capsules on both the lateral and lower sides of the breast compared with other regions. Most of the patients did not go through surgical procedures and were treated with tranilast to resolve capsular contracture. Among patients who underwent surgical procedures, recurrence of capsular contracture was observed in two cases. In these two cases, an improvement in the degree of capsular contracture was observed: grade IV according to the Baker–Spear classification system before surgery and grade III in both cases postoperatively.

## Discussion

Our study distinguishes from other studies in that while most studies focus on resolving capsular contracture in breast augmentation, we focused on its treatment in breast reconstruction. Capsular contracture in breast reconstruction negatively impacts a patient's quality of life in that it causes physical discomfort or pain, as the capsule tightens around the implant. This can lead to a distorted breast shape, resulting in aesthetic dissatisfaction. Additionally, the tightness and discomfort can restrict movement, making daily activities more difficult. The need for corrective surgeries also adds emotional and financial stress, further diminishing overall well-being. Therefore, understanding the mechanisms behind capsular contracture is crucial not only for improving patients' overall satisfaction with breast reconstruction but also for ensuring the success of the reconstruction itself.

When an implant is inserted into the body, it is recognized as a foreign substance, causing an inflammatory response and creating a fibrous capsule that encloses the implant. The capsule created this way usually has a benign character, but in some cases, it causes additional inflammatory reactions, compressing the implant, and causing pain and deformation of the breast shape.

Factors associated with the etiology of capsular contracture include a history of postoperative radiation and the presence of chronic inflammation, such as chronic seroma and subclinical bacterial infection. Although there is a consensus among surgeons on the inflammatory nature of capsular fibrosis, the diverse array of inciting events initiating the inflammatory cascade renders it challenging to predict why some patients develop capsular contracture while others do not. 14 Seroma, which is a potent medium for bacterial proliferation and known to harbor a notably high concentration of proinflammatory cytokines, poses an elevated risk of capsular contracture. This heightened risk stems from increased levels of proinflammatory mediators within the periprosthetic capsule, which drives fibrosis. Additionally, residual seroma fluid, rich in inflammatory mediators, may predispose to bacterial infection and biofilm formation. 15

History of radiation therapy is also known for its effect on capsular contracture by inducing capsule contracture and fibrosis of surrounding tissues. Both prepectoral and subpectoral breast reconstructions exhibit elevated rates of capsular contracture with a history of radiation therapy. 16 Histological studies highlight heightened elastin and cellular infiltrates in native capsules with a radiation therapy history, emphasizing its involvement in capsular contracture development. 17

Although age is not known to be a factor related to capsular contracture, the significant difference observed in this study may be attributed to the high proportion of younger patients among Korean breast cancer patients. It is generally known that collagen synthesis decreases with age. 18 19 It can be inferred that older patients have relatively reduced collagen synthesis activity, which in turn might influenced decreased incidence of capsular contracture.

Through multivariate analysis, which was adjusted for many confounders, our study showed that the presence of seroma and a history of radiation therapy was an independent correlating factor of capsular contracture. This finding supports the idea that the presence of a seroma, rich in inflammatory mediators, is an independent correlating factor of capsular contracture. When we examined our cases of partial capsulectomy for capsular contracture, capsular contracture was observed mainly in the dependent portion of the body, the location where the seroma was likely situated, further supporting this idea.

While capsulectomy is commonly recommended for managing capsular contracture, a recent meta-analysis found no significant difference in recurrence rates between capsulectomy and capsulotomy. 20 Also, autologous reconstruction is often considered the definitive solution due to its elimination of long-term risks and more natural results, our findings suggest that partial capsulectomy could be a viable alternative. 21 In our study, partial capsulectomy in 18 cases achieved a resolution rate of approximately 90%, suggesting this approach may offer an effective solution for capsular contracture in breast reconstruction.

Regarding the limitations of our study, this was a single-center, retrospective study with a limited number of patients who underwent partial capsulectomy, and the results would have been more accurate and powerful with a larger sample size. Therefore, further studies with larger sample sizes are required to validate and generalize our findings. Also, due to the retrospective nature of this study, the evaluation of Baker grade was conducted by the operator during outpatient visits. This evaluation may hinder the objectivity of the study in that when assessing capsular contracture, the evaluation can vary among observers. Therefore, the frequency or stage of capsular contracture may differ based on who is conducting the assessment. 22 The absence of alternative objective evaluation standards has left us with no choice but to use such methods as most studies on capsular contracture do so.

### Conclusion

Our multivariate analysis demonstrated that age, seroma, rippling, and radiotherapy were independent correlating factors for capsular contracture in prepectoral DTI. Partial capsulectomy, a relatively simple and less invasive option, could be considered as an alternative to traditional capsulectomy or autologous tissue reconstruction for managing capsular contract. Through our study, we hope to pave the way for larger, more objective research on this topic, ultimately contributing to the establishment of guidelines for the surgical management of capsular contracture in the future.
